# NK and T Cell Differentiation at the Maternal-Fetal Interface in Sows During Late Gestation

**DOI:** 10.3389/fimmu.2020.582065

**Published:** 2020-09-11

**Authors:** Melissa R. Stas, Michaela Koch, Maria Stadler, Spencer Sawyer, Elena L. Sassu, Kerstin H. Mair, Armin Saalmüller, Wilhelm Gerner, Andrea Ladinig

**Affiliations:** ^1^University Clinic for Swine, Department for Farm Animals and Veterinary Public Health, University of Veterinary Medicine Vienna, Vienna, Austria; ^2^Institute of Immunology, Department of Pathobiology, University of Veterinary Medicine Vienna, Vienna, Austria; ^3^Christian Doppler Laboratory for Optimized Prediction of Vaccination Success in Pigs, Institute of Immunology, Department of Pathobiology, University of Veterinary Medicine Vienna, Vienna, Austria

**Keywords:** porcine placenta, CD4 T cells, CD8 T cells, natural killer cells, late gestation, flow cytometry

## Abstract

The phenotype and function of immune cells that reside at the maternal-fetal interface in humans and mice have been, and still are, extensively studied with the aim to fully comprehend the complex immunology of pregnancy. In pigs, information regarding immune cell phenotypes is limited and mainly focused on early gestation whereas late gestation has not yet been investigated. We designed a unique methodology tailored to the porcine epitheliochorial placenta, which allowed us to address immune phenotypes separately in the maternal endometrium (ME) and fetal placenta (FP) by flow cytometry. In-depth phenotyping of NK cells, non-conventional and conventional T cells within maternal blood (mBld), ME, FP, and fetal spleen (fSpln) revealed major differences between these anatomic sites. In both maternal compartments, all NK cells were perforin^+^ and had NKp46-defined phenotypes indicative of late-stage differentiation. Likewise, T cells with a highly differentiated phenotype including CD2^+^CD8α^+^CD27^dim/–^perforin^+^ γδ T cells, CD27^–^perforin^+^ cytolytic T cells (CTLs), and T-bet^+^ CD4^+^CD8α^+^CD27^–^ effector memory T (Tem) cells prevailed within these compartments. The presence of highly differentiated T cells was also reflected in the number of cells that had the capacity to produce IFN-γ. In the FP, we found NK cells and T cell populations with a naive phenotype including CD2^+^CD8α^–^CD27^+^perforin^–^ γδ T cells, T-bet^–^CD4^+^CD8α^–^CD27^+^ T cells, and CD27^+^perforin^–^ CTLs. However, also non-naive T cell phenotypes including CD2^+^CD8α^+^CD27^+^perforin^–^ γδ T cells, T-bet^+^CD4^+^CD8α^+^CD27^–^ Tem cells, and a substantial proportion of CD27^–^perforin^+^ CTLs resided within this anatomic site. Currently, the origin or the cues that steer the differentiation of these putative effector cells are unclear. In the fSpln, NKp46^high^ NK cells and T cells with a naive phenotype prevailed. This study demonstrated that antigen-experienced immune cell phenotypes reside at the maternal-fetal interface, including the FP. Our methodology and our findings open avenues to study NK and T cell function over the course of gestation. In addition, this study lays a foundation to explore the interplay between immune cells and pathogens affecting swine reproduction.

## Introduction

A successful pregnancy builds upon two aspects of the maternal immune system that need to be balanced. On the one hand, the maternal immune system needs to tolerate the semi-allogeneic fetus, but at the same time it should also be able to detect and respond to local pathogens in order to protect the fetus. This is coordinated by cells of the innate and adaptive immune systems but also the decidual microenvironment (1). Immune cells in the decidua vary in composition, phenotype and function and change with the stage of gestation (2, 3). During human pregnancy, ∼40% of the decidual stromal cells can be characterized as CD45^+^ leukocytes (4, 5). Blastocyst implantation is characterized by the upregulation of inflammatory genes (6), production of pro-inflammatory cytokines, and immune cell recruitment (2). The following immune cells populate the first trimester human decidua: uterine natural killer (uNK) cells (∼70%), macrophages (∼20%), and T cells (∼5–20%) (7–9). In addition, dendritic cells, mast cells, and B cells are present, but in low frequencies (10, 11). Toward term, the frequency of uNK cells in human decidua diminishes whereas the T cell frequency increases (5, 9). Dynamic changes in immune cell composition also occur over the course of murine gestation (12). Altogether, cooperation of various immune cells and dynamic changes are a prerequisite of successful pregnancy.

The prominent population of uNK cells, found in humans, can be identified by a CD56^*bright*^CD16^–^KIR^+^CD9^+^CD49a^+^ phenotype (13). These uNK cells have a poor cytolytic activity despite the fact that they contain ample granules filled with cytolytic machinery (13). Their main task during pregnancy is to produce a wide range of cytokines, proangiogenic factors, and proteases by which they are involved in vascular remodeling, neovascularization, and fetal tolerance (10, 14, 15). Apart from their essential role in maintaining fetal tolerance, it has recently been shown that uNK cells can also effectively combat potential viral threats in the decidua (16, 17).

Moreover, conventional TCR-αβ^+^ and a sparse population of non-conventional TCR-γδ^+^ T cells populate the decidua during gestation (18, 19). In humans, decidual CD8^+^ T cells account for ∼45–75% whereas decidual CD4^+^ T cells only account for ∼30–45% (4, 8, 18, 19). These frequencies seem to remain constant over time, however, one study showed an increase of CD4^+^ T cells at term which seems to play a role in parturition (20). Furthermore, many studies have shown that for both T cell subsets an antigen-experienced phenotype, based on CD45RA/CD45RO or CD45RA/CCR7 phenotypes, prevails (21–23). Decidual CD8^+^ (dCD8) T cells are competent producers of cytokines and show cytolytic activity (24). At term, dCD8^+^ T cells seem to be activated but have reduced protein expression of perforin and granzyme B (22, 25). Recently, it has been shown that the translation of cytolytic molecules is blocked (17), and that this blockage might be lifted by pro-inflammatory events (26). CD4^+^ T cell subsets, or T helper cells (Th), can be categorized based on their cell surface expression of chemokine receptors (CCR6, CCR4, and CXCR3) and intracellular expression of specific transcription factors. In the human decidua Th1, Th2, and Th17 cells constitute about ∼30, ∼5, and 2–5%, respectively (8, 27). It has been shown that decidual T cells with viral specificity might provide fetal protection (28). Specificity for fetal/paternal antigens has also been demonstrated (29–31), so in this context it is crucial that potential aberrant responses are contained. One of the mechanisms suppressing T cell effector functions is mediated by regulatory T (Treg) cells with/without fetal/paternal specificity (29, 30, 32, 33). Indeed, in humans and mice, CD4^+^CD25^high^Foxp3^+^ cells comprise about 5–20% of the decidual CD4^+^ T cells (8, 9, 30, 34).

Pigs have an epitheliochorial type of placenta as defined by the presence of two epithelial layers that compartmentalize the maternal and fetal component. Due to this special anatomy, the porcine placenta is considered as a tight barrier through which the transfer of maternal antibodies is impossible (35). Hence, it is assumed that the porcine placenta is also impermeable to cells. Nevertheless, some pathogens like the porcine reproductive and respiratory syndrome virus (PRRSV) can breach this barrier and infect the porcine fetuses. If these infections occur during late gestation, abortions are a frequent outcome. The immune cells populating the porcine placenta during health and disease have not been studied in detail so far. It has been reported that NK cells and T cells can be identified in the endometrium of pregnant pigs during early gestation, however, only major immune cell subsets were characterized (36–38).

In this study, we aimed to investigate immune cell subsets in the porcine placenta and their phenotypes related to functional traits in detail. We exploited the feature of an epitheliochorial placenta and established a separation and leukocyte isolation procedure in order to study immune cells from the maternal endometrium (ME) and fetal placenta (FP). We focused on NK and T cell phenotypes due to their abundance in the human and murine placenta but also to lay foundation for future studies addressing the functionality and role of these cells during viral infections in the porcine reproductive tract.

## Materials and Methods

### Animals and Sample Collection

Three healthy multiparous crossbred (Landrace × Large White) pregnant sows were obtained from a commercial Austrian piglet producing farm, unsuspicious for PRRSV, confirmed by regular serological monitoring. Sows are routinely vaccinated against porcine parvovirus in combination with *Erysipelothrix rhusiopathiae* and swine influenza virus. The age of the sows (sow No. 2, 3.3 years; and sow No. 3, 2.7 years) were determined based on the date of birth and date of scheduled euthanasia. Unfortunately, we were unable to determine the age of sow No. 1. The sows and their litters (gestation days >100) were anesthetized by intravenous injection of Ketamine (Narketan^®^ 100 mg/mL, Vetoquinol Österreich GmbH, Vienna, Austria, 10 mg/kg body weight) and Azaperone (Stresnil^®^ 40 mg/mL, Elanco GmbH, Cuxhaven, Germany, 1.5 mg/kg body weight) during late gestation. Maternal blood (mBld) was taken by cardiac puncture and transferred into collection cups containing heparin. Afterward, animals were euthanized via intracardial injection of T61^®^ (Intervet GesmbH, Vienna, Austria, 1 mL/10 kg body weight). The abdomen of the sows was incised and the complete uterus was removed and placed in a trough. Uteri were opened at the anti-mesometrial side. Per sow, three average sized fetuses were randomly selected and removed with their umbilical cord, placenta and a portion of uterus adjacent to the umbilical stump. The abdomen of each fetus was opened in order to collect the intact fetal spleen (fSpln) in collection cups containing phosphate-buffered saline (PBS, PAN-Biotech, Aidenbach, Germany). For collection of the maternal-fetal interface of each fetus, the myometrium was trimmed off and the ME and FP were mechanically separated by the use of forceps. Approximately 80 g of ME and 90 g of FP were collected and transferred into RPMI-1640 with stable L-glutamine supplemented with 100 IU/mL penicillin and 0.1 mg/mL streptomycin (all from PAN-Biotech). During pathological examination of the sows, no pathologic lesions were found and their litters were normal. Since all procedures were done on dead animals, no federal animal ethics approval was required according to Austrian law. The project plan has been discussed and approved by the institutional ethics and animal welfare committee in accordance with GSP guidelines and national legislation (approval number ETK-32/02/2016).

### Cell Isolation

Peripheral blood mononuclear cells (PBMCs) were procured from heparinized maternal blood via density gradient centrifugation (Pancoll human, density 1.077 g/mL, PAN-Biotech, 30 min at 920 × *g*). fSplns were kept on ice and cut into small pieces. The tissue was further dissociated by sieving it through a coarse-meshed sieve, which was regularly rinsed with cold PBS (PAN-Biotech). Collected cells were washed by centrifugation and after resuspension in PBS filtered through a Corning^®^ 70 μm cell strainer (Falcon, BD Biosciences, San Jose, CA, United States). The obtained cell suspension was subjected to density gradient centrifugation under the conditions described above. Tissues from ME and FP were cut into small pieces and digested in RPMI-1640 supplemented with 2% (v/v) heat-inactivated fetal calf serum (FCS, Sigma-Aldrich, Schnelldorf, Germany), 25 U/mL DNase type I (Thermo Fisher Scientific, Carlsbad, CA, United States), 300 U/mL Collagenase Type I (Thermo Fisher Scientific), 100 IU/mL penicillin (PAN-Biotech) and 0.1 mg/mL P/S streptomycin (PAN-Biotech) for 1 h at 37°C during constant shaking. Obtained cell suspensions were drained through a coarse-meshed sieve and the flow-through was filtered through cotton wool to eliminate dead cells. Cells were resuspended in 40% Percoll (13 mL, Thermo Fisher Scientific), underlaid with 70% Percoll (13 mL) and subjected to density gradient centrifugation under the same conditions as described before. Isolated cells from mBld, fSpln, ME, and FP were subjected to three consecutive washing steps (350 × *g*, 10 min, 4°C): first with PBS, followed by two washes with RPMI-1640 (first wash 5% FCS, second wash 10% FCS, all other supplements as described above). Thereafter, cells were immediately used for immune phenotyping or subjected to IFN-γ enzyme-linked immune absorbent spot (ELISpot) assays.

### Flow Cytometry Staining

A detailed overview of the mAbs and secondary reagents used for flow cytometry (FCM) staining is given in Table 1. A total of 2 × 10^6^ cells were plated out in a round-bottom 96-well microtiter plate (Greiner Bio-One, Frickenhausen, Germany) and were stained in a six or seven step-procedure. After each incubation step (20 min, 4°C) the cells were washed twice with 200 μl PBS + 10% (v/v) porcine plasma (in-house preparation) or as indicated. Surface markers were stained with biotinylated or non-conjugated primary mAbs followed by isotype-specific secondary antibodies or streptavidin conjugates. This was followed by incubation with whole mouse IgG molecules (2 μg/sample, ChromPure, Jackson ImmunoResearch, West Grove, PA, United States) in order to block free binding sites of mouse-isotype specific secondary antibodies. In a further incubation step, the Fixable Viability Dye eFluor 780 (Thermo Fisher Scientific) was applied according to the instructions of the manufacturer. During this incubation, samples were also labeled with directly conjugated mAbs or biotinylated antibodies. For the CD4 and CD8β T cell samples (Table 1) this was followed by incubation with the streptavidin conjugate BV510 (BioLegend, San Diego, CA, United States). Samples were fixed and subsequently permeabilized with the Foxp3/Transcription Factor Staining Buffer Set (Thermo Fisher Scientific) according to the manufacturer’s instructions. Finally, an intracellular staining for transcription factors or perforin was performed. For NK cells, γδ T cells, and CD8 T cells fluorescence minus one (FMO) control samples without perforin, GATA-3, and CD8β were prepared, respectively.

**TABLE 1:** Antibodies and streptavidin-conjugates used for FCM staining.

Marker	Clone	Isotype	Source	Labeling	Fluorophore
**Leukocyte characterization**					
CD45	K252.1E4	IgG1	Bio-Rad	Direct	Alexa647
**NK cells and CD16^+^ T cells**					
CD3	PPT3	IgG1	In-house	Indirect^A^	PerCP-eFluor710
CD8α	11/295/33	IgG2a	In-house	Indirect^B^	BV510
CD172a	74-22-15A	IgG2b	In-house	Indirect^C^	BV421
NKp46	VIV-KM1	IgG1	In-house	Direct	Alexa647
CD16	G7	IgG1	Bio-Rad	Direct	FITC
Perforin	δ-G9	IgG2b	eBioscience	Direct	PE
**γδ T cells**					
TCR-γδ	PPT16	IgG2b	In-house	Indirect^C^	BV421
CD8α	11/295/33	IgG2a	In-house	Indirect^B^	BV510
CD2	MSA4	IgG2a	In-house	Direct	Alexa488
CD27	b30c7	IgG1	In-house	Direct	Alexa647
GATA-3	TWAJ	IgG2b	eBioscience	Direct	PerCP-eFluor710
Perforin	δ-G9	IgG2b	eBioscience	Direct	PE
**CD4^+^ T cells**					
CD4	74-12-4	IgG2b	In-house	Indirect^D^	Alexa488
CD8α	11/295/33	IgG2a	In-house	Indirect^E^	PerCP-eFluor710
CD25	3B2	IgG1	In-house	Indirect^F^	BV421
CD27	b30c7	IgG1	In-house	Direct	Alexa647
CD3	PPT3	IgG1	SBA	Indirect^B^	BV510
Foxp3	FJK-16s	IgG2a	eBioscience	Direct	PE
T-bet	4B10	IgG1	eBioscience	Direct	PE
**CD8^+^ T cells**					
CD8β	PPT23	IgG1	In-house	Indirect^G^	Alexa488
CD8α	11/295/33	IgG2a	In-house	Indirect^B^	BV510
CD3	BB23-8E6	IgG2b	SBA	Indirect^C^	BV421
CD27	b30c7	IgG1	In-house	Direct	Alexa647
Perforin	δ-G9	IgG2b	eBioscience	Direct	PE

*^*A*^Rat-anti-mouse anti-IgG1-PerCPefluor710, eBioscience, ^*B*^Streptavidin-BV510, BioLegend, ^*C*^Goat-anti-mouse anti-IgG2b-BV421, Jackson Immuno Research, ^*D*^Goat-anti-mouse anti-IgG2b-Alexa488, Jackson Immuno Research, ^*E*^Rat-anti-mouse anti-IgG2a-PerCPefluor710, ^*F*^Rat-anti-mouse anti-IgG1-BV42, BioLegend, ^*G*^Goat-anti-mouse anti-IgG1-Alexa488, Thermo Fisher Scientific.*

### FCM Analysis

Flow cytometry samples were measured on a CytoFLEX LX (Beckman Coulter GmbH, Krefeld, Germany) flow cytometer equipped with six lasers (375, 405, 488, 561, 638, and 808 nm). For all samples, at least 1 × 10^5^ lymphocytes were recorded. Single stains were performed using the VersaComp antibody capture kit (Beckman Coulter GmbH, Krefeld, Germany), according to the manufacturer’s instructions, and compensation values were calculated by CytExpert software version 2.3.1.22 (Beckman Coulter). Further data processing was completed by FlowJo software version 10.5.3 (BD Biosciences). A consecutive gating strategy was applied for the phenotypic characterization of the isolated cells (Supplementary Figure 1). First, a time gate was applied and lymphocytes were selected according to their light scatter properties (FSC-A vs. SSC-A) and were subjected to doublet discrimination (FSC-H vs. FSC-A and SSC-H vs. SSC-A). Hereafter, viable cells were gated using the fixable viability dye eFluor780^®^ and cells with a high auto fluorescent signal were excluded by using a bandpass filter 610/20 in the excitation line of the blue laser.

### IFN-γ ELISpot Assay

Cells isolated from mBld, ME, FP, and fSpln were subjected to IFN-γ ELISpot assays. Coating and development of ELISpot plates was performed as described (39), with the only difference that for detection the biotinylated mouse anti-porcine IFN-γ clone P2C11 (Mabtech, Nacka Strand, Sweden) was used at a concentration of 0.125 μg/mL. Per well, 3 × 10^5^ cells were plated in cell culture medium (RPMI1640 with 10% FCS, other ingredients as above). To induce IFN-γ production, cells were stimulated with Staphylococcal enterotoxin B (SEB, 500 ng/mL, Sigma-Aldrich). Cells from each location were plated in duplicates for 24 h at 37°C and 5% CO_2_. Spots were counted with an AID ELISpot reader (AID, Straßberg, Germany).

### Statistical Analysis and Data Representation

The frequencies of cell lineages, expressed within viable lymphocytes, were exported to Microsoft Excel (Office 2016, Microsoft, Redmond, WA, United States) and were corrected for CD45 expression by multiplying the percentage with the CD45 correction factor. The CD45 correction factor was calculated, for each individual sample, by dividing 100% by the percentage of CD45^+^ cells. Data processed in FlowJo and Microsoft Excel were imported into Graphpad Prism version 8.1.0 (GraphPad Software Inc., San Diego, CA, United States) for descriptive analysis and graphical representation. For each anatomic location and for each cell population the mean and individual values are given. For the IFN-γ producing cells in the ELISpot assay results of each duplicate are shown as the mean ± standard error of the mean (SEM).

## Results

### Identification and Frequency of CD45^+^ Lymphocytes

**FIGURE 1:**
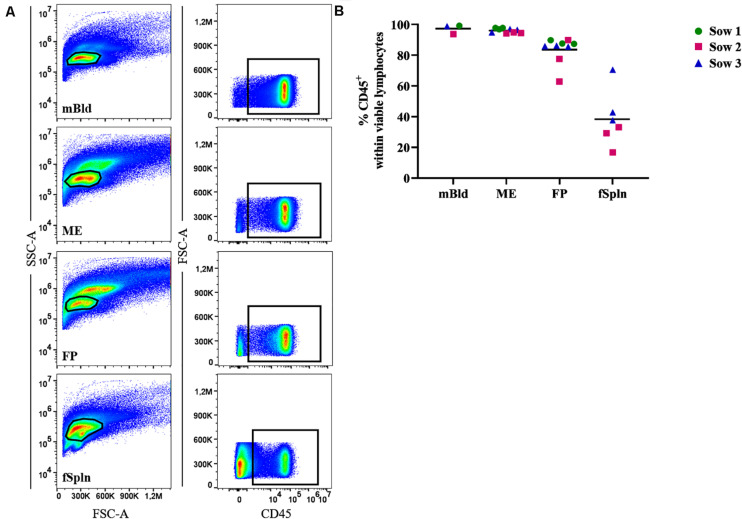
Frequency of CD45^+^ lymphocytes in mBld, ME, FP and fSpln. **(A)** Lymphocytes were gated based on their SSC-A vs. FSC-A properties and after consecutive gating to exclude doublets, dead cells and cells with high autofluorescence (see Supplementary Figure 1), CD45^+^ cells were gated. Representative pseudocolor plots for maternal blood (mBld), maternal endometrium (ME), fetal placenta (FP), and fetal spleen (fSpln) are shown. **(B)** Frequency of CD45^+^ cells within viable lymphocytes. Each colored symbol represents data from one sow for mBld (*n* = 3) or fetuses coming from that sow for ME (*n* = 9), FP (*n* = 9), and fSpln (*n* = 6). The black bars display the mean within the respective anatomic location.

Leukocyte isolation procedures for ME and FP based on enzymatic tissue digestion and subsequent gradient centrifugation were established for this study. To evaluate the performance of this procedure, we initially investigated the presence of total lymphocytes in the obtained cell preparations by studying the cell surface expression of CD45 (leukocyte common antigen) in a two-parameter FCM staining (Figure 1). We investigated the frequency of CD45^+^ cells in our predefined lymphocyte gate, as shown by a basic gating overview, for mBld, ME, FP, and fSpln (Figure 1A). An overview of the complete consecutive gating strategy for all investigated locations is provided in Supplementary Figure 1. Collective data of CD45^+^ cells for the investigated anatomic locations are displayed in Figure 1B. In mBld and ME, over 95% of cells within our population of viable cells expressed CD45. In FP, with the exception of two individual fetuses (62.8% and 77.5%), on average 84.1% of the viable lymphocytes expressed CD45. For cells isolated from the fSpln the frequency of CD45^+^ cells within the population of viable lymphocytes varied with a range of 16.3–70.8%. Hence, the vast majority of cells in our predefined lymphocytes gate were CD45^+^ cells for the investigated locations, with the exception of fSpln. In embryos, fetuses, and neonates, the spleen is also capable of hematopoiesis; therefore, it is conceivable that the CD45^–^ cells represent immature stem cells that will acquire CD45 during their maturation process (40). Accordingly, with the obtained CD45 frequencies a CD45-correction factor was calculated and used to determine the distribution of the major NK and T cell frequencies thereafter (see also section “Statistical Analysis and Data Representation”).

### Characterization of NK Cells

**FIGURE 2:**
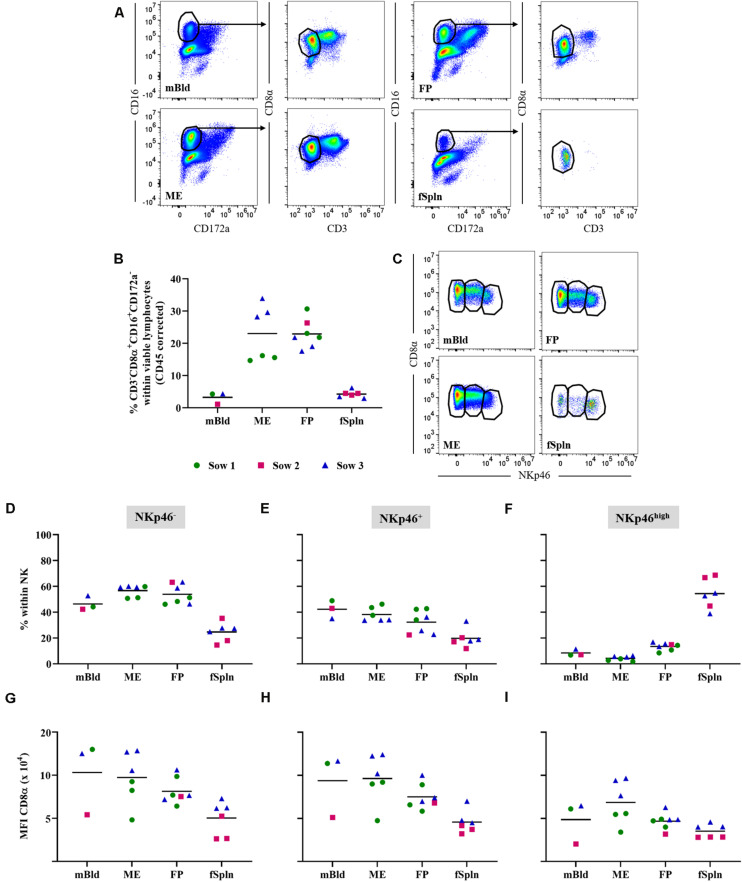
Characterization of NK cells. **(A)** Following exclusion of doublets, dead cells, and cells with high autofluorescence (see Supplementary Figure 1), total NK cells were identified by gating on CD16^+^CD172a^–^ cells and further subgated for CD3^–^CD8α^+^ cells. Representative pseudocolor plots for maternal (left) and fetal compartments (right) are shown. **(B)** Frequencies of CD3^–^CD8α^+^CD16^+^CD172a^–^ cells (total NK cells) within viable lymphocytes corrected for CD45 expression. **(C)** Total NK cells in the different anatomic locations were gated to identify three NK cell subsets: CD8α^+^NKp46^–^, CD8α^+^NKp46^+^, and CD8α^dim^NKp46^high^ (from left to right). **(D–F)** Distribution of CD8α/NKp46-defined NK cell subsets within the respective anatomic locations. **(G–I)** CD8α/NKp46-defined NK cells subsets were further analyzed for their CD8α expression levels. The median fluorescence intensity (MFI) for CD8α within the respective NK cell subsets is shown. **(B,D–I)** For all graphs, each colored symbol represents data from one sow for mBld (*n* = 3) or fetuses coming from that sow for ME (*n* = 6), FP (*n* = 7), and fSpln (*n* = 6). The black bars display the mean within the respective anatomic location.

Porcine NK cells can be defined by their perforin^+^CD3^–^CD8α^+/dim^CD16^+^CD172a^–^NKp46^+/–^ phenotype (41–43). Following FCM staining, we used a CD3^–^CD8α^+^CD16^+^CD172a^–^ phenotype to identify the total NK cell population in the investigated anatomic sites during late gestation (Figure 2A). An enrichment of total NK cells in the ME (mean: 23%) and the FP (mean: 22.8%) as opposed to their frequency in mBld and fSpln (mean: 3.2 and 4.2%) was found (Figure 2B). CD3^–^CD8α^+^CD16^+^CD172a^–^ NK cells were further analyzed for their expression of NKp46 (CD335; NCR1). This enabled us to identify NKp46^–^, NKp46^+^, and NKp46^high^ expressing NK cells in mBld, ME, FP, and fSpln (Figure 2C). Collective data with regard to the distribution of the NKp46-defined populations are summarized in Figures 2D–F. Both NKp46^–^ and NKp46^+^ NK cells could be identified in mBld, ME, and FP. Overall, in mBld NKp46^–^ and NKp46^+^ NK cells were represented in equal numbers (46.4 and 42.3%) whereas in ME and FP their distribution was on average 56.7 and 53.9% vs. 38.1 and 32.3%. Within fSpln all three NK cell populations were found to be present, however, the NKp46^high^ subset predominated (mean: 54.4%). In addition, CD8α expression levels in the three NKp46-defined NK cell subsets were investigated (Figures 2G–I) and highlighted similarities and dissimilarities between the three NK cell subsets. NK cells with a NKp46^–^ and NKp46^+^ phenotype consistently showed comparable CD8α expression levels (Figures 2F,G) whereas NKp46^high^ NK cells showed the lowest expression of CD8α as reflected by the MFI levels (Figure 2I). In order to complete the NK cell phenotype, we addressed the intracellular expression of the cytolytic molecule perforin (Supplementary Figure 2). It was found that all NK cells expressed perforin within all investigated anatomic locations.

### Characterization of Lymphocytes With Phenotypic Features of NK and T Cells

**FIGURE 3:**
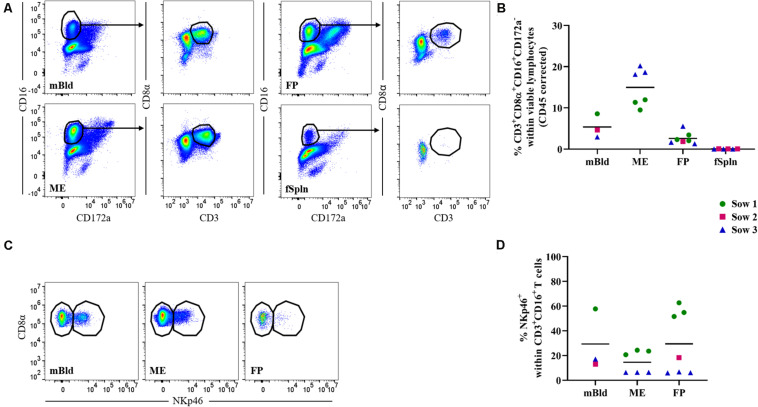
Characterization of lymphocytes with phenotypic features of NK and T cells. **(A)** Following exclusion of doublets, dead cells, and cells with high autofluorescence (see Supplementary Figure 1), CD16^+^ T cells were identified by gating on CD16^+^CD172a^–^ cells and further subgated on CD3^+^CD8α^+^ cells. Representative pseudocolor plots for maternal (left) and fetal compartments (right) are shown. **(B)** Frequencies of non-conventional CD3^+^CD8α^+^CD16^+^CD172a^–^ cells within viable lymphocytes corrected for CD45 expression. **(C)** CD16^+^ T cells in mBld, ME, and FP were analyzed for their NKp46 surface expression and separated in CD8α^+^NKp46^–^ and CD8α^+^NKp46^+^ CD16^+^ T cells. **(D)** Frequency of CD8α^+^NKp46^+^ within CD16^+^ T cells in mBld, ME, and FP. **(B,D)** For all graphs, each colored symbol represents data from one sow for mBld (*n* = 3) or fetuses coming from that sow for ME (*n* = 6), FP (*n* = 7), and fSpln (*n* = 6). The black bars display the mean within the respective anatomic location.

Within our NK cell sample, a CD3^+^CD8α^+^CD16^+^CD172a^–^ lymphocyte population could be identified in all anatomic sites with the exception of the fSpln (Figure 3A). Interestingly, this phenotype constituted between 9.5 and 20.2% of total viable lymphocytes in the ME while the abundance of this phenotype in mBld (<5.4%) and FP (<2.6%) was lower (Figure 3B). We further analyzed the CD3^+^CD8α^+^CD16^+^CD172a^–^ lymphocytes for their surface expression of NKp46 and intracellular expression of perforin in a similar manner to the NK cells. Due to the absence of this phenotype in the fSpln, no data on NKp46 or perforin expression is shown for this anatomic location. In mBld and at the maternal-fetal interface, a substantial proportion of CD3^+^NKp46^+^ lymphocytes could be observed (Figures 3C,D). However, we did observe a high degree of sow-to-sow variation which was most pronounced in the FP where the average frequency of NKp46^+^ cells within CD3^+^ T cells for fetuses from one sow (No. 1) was 56.3% and for those from sow No. 3 was 6.3% (Figure 3D). Furthermore, all CD3^+^CD8α^+^CD16^+^CD172a^–^lymphocytes were positive for perforin (Supplementary Figure 3), and therefore might be capable of cytolytic activity.

### Characterization of γδ T Cells

**FIGURE 4:**
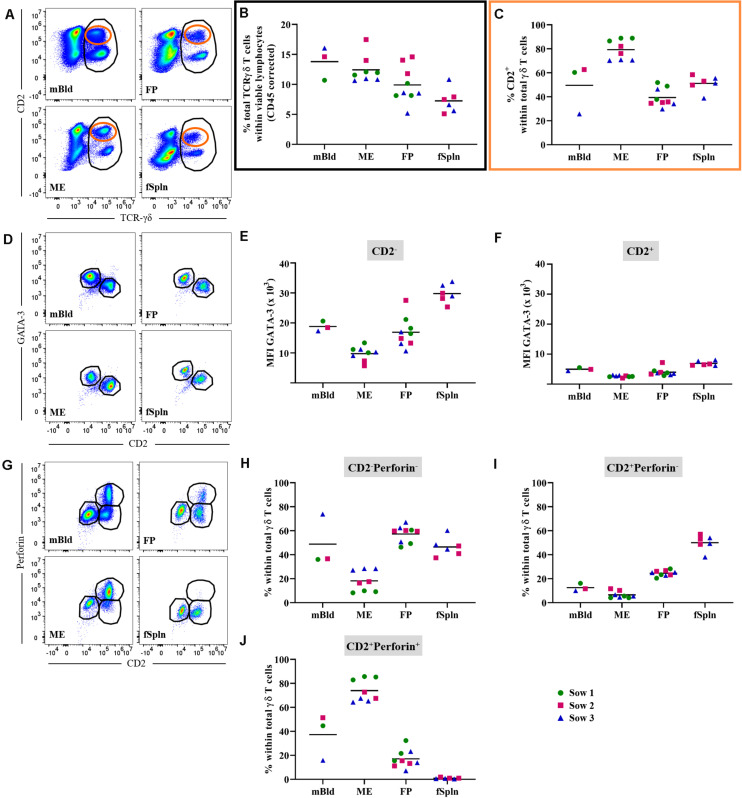
Characterization of γδ T cells. **(A)** Following exclusion of doublets, dead cells, and cells with high autofluorescence (see Supplementary Figure 1), total γδ T cells at all four anatomic locations were gated (black gate) based on their TCR-γδ expression. The γδ T cells were further subgated based on their CD2^+^ phenotype (orange gate). Representative pseudocolor plots for maternal (left) and fetal compartments (right) are shown [applies also for **(D,G)**]. **(B)** Frequencies of total TCR-γδ T cells within viable lymphocytes corrected for CD45 expression. **(C)** Frequency of CD2^+^ TCR-γδ T cells within total γδ T cells. **(D)** Analysis of total γδ T cells for their expression of CD2 and GATA-3. CD2^–^GATA3^+^ subsets and CD2^+^GATA-3^–/low^ subsets were gated. **(E,F)** Median fluorescence intensity (MFI) of GATA-3 in CD2^–^ **(E)** and CD2^+^ **(F)** γδ T cells at the four anatomic locations. **(G)** Analysis of total γδ T cells for their expression of CD2 and perforin. CD2^–^perforin^–^, CD2^+^perforin^–^, and CD2^+^perforin^+^ population were gated. CD2^+^perforin^+^ γδ T cells could not be identified in fetal spleen (fSpln). **(H–J)** Distribution of the three CD2/perforin-defined γδ T cell subsets within the respective anatomic locations. **(B,C,E,F,H–J)** For all graphs, each colored symbol represents data from one sow for mBld (*n* = 3) or fetuses coming from that sow for ME (*n* = 8), FP (*n* = 9), and fSpln (*n* = 6). The black bars display the mean within the respective anatomic location.

Porcine γδ T cells can be characterized by a set of surface molecules including CD2, CD8α, and CD27, as well as intracellular molecules including transcription factors and cytolytic molecules (44). We aimed to identify the total γδ T cell population by targeting a γδ-specific CD3 molecule by using monoclonal antibody clone PPT16 (45), as depicted in Figure 4A (black gate). The mean frequencies of total γδ T cells varied slightly between the investigated anatomic locations (Figure 4B; black box), with the highest abundance in mBld (mean: 13.9%) followed by 12.8% in ME, 10.2% in FP, and the lowest frequency was found in the fSpln (mean: 7.8%). Following the characterization of total γδ T cells we analyzed their expression of CD2, intending to determine the CD2^+^/CD2^–^ γδ T cell ratio within the investigated locations (Figure 4A; orange gate). Both γδ T cell subsets were found and revealed striking differences between the four investigated anatomic locations. Results procured from all investigated locations are visualized in Figure 4C. The ratio of CD2^+^ to CD2^–^ γδ T cells in mBld and fSpln was on average 1:1. The ME was particularly enriched for CD2^+^ γδ T cells (79.3%) whilst the FP was predominantly colonized by CD2^–^ γδ T cells (39.3% CD2^+^, 60.7% CD2^–^) (Figure 4C).

Additionally, we explored the expression of CD2 in association with GATA-3 or perforin within all investigated anatomic sites. We were able to detect a subset of CD2^–^GATA-3^+^ and CD2^+^GATA-3^low/–^ γδ T cells within all four locations (Figure 4D). To demonstrate the differences in GATA-3 expression levels between the two γδ subsets, we calculated the MFI for both subsets within the investigated locations. The results for the MFI for all samples analyzed for both γδ subsets are summarized in Figures 4E,F. For all investigated locations, CD2^–^ γδ T cells showed consistently a higher expression of GATA-3 (Figure 4E) as compared to their CD2^+^ counterpart (Figure 4F). However, CD2^–^ γδ T cells isolated from the ME displayed the lowest expression of GATA-3 (MFI ranging from 5681 to 13,338) while the highest expression was observed in fSpln (MFI varied from 25,330 to 33,773) (Figure 4E). The expression pattern of CD2 in association with perforin allowed us to identify CD2^–^perforin^–^, CD2^+^perforin^–^, and CD2^+^perforin^+^ γδ T cells within all investigated locations except the fSpln (Figure 4G). The composition of the CD2/perforin-defined γδ T cell subsets diverged between the anatomic locations (Figures 4H–J). The frequency of CD2^–^perforin^–^ γδ T cells was the lowest in the ME (mean: 18.1%) and highest in FP (mean: 57.3%). In mBld and fSpln this phenotype constituted on average 48.8 and 46.4% of the γδ T cells, respectively. γδ T cells with a CD2^+^perforin^–^ phenotype were highest in fSpln (mean: 50%) whereas lower frequencies were found in FP, mBld, and ME (24.5, 12.5, and 6.5%, respectively). Furthermore, phenotyping revealed that the ME was enriched with CD2^+^perforin^+^, putative cytolytic effector γδ T cells, which constituted about 74% of total γδ T cells. In the fSpln this phenotype could not be observed. Interestingly, this phenotype of putative effector cells was also found in the FP (mean: 17%) and mBld (mean: 37.3%).

**FIGURE 5:**
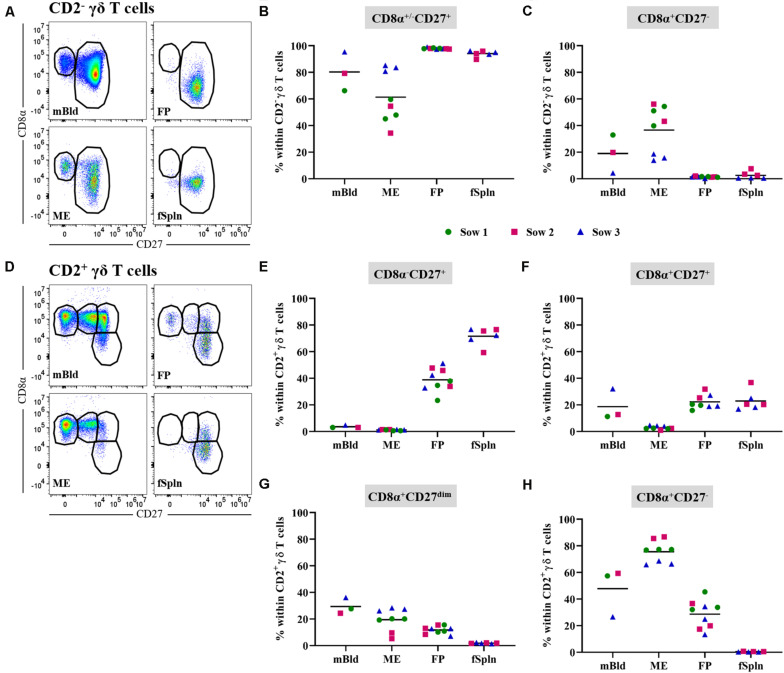
CD8α/CD27 expression on CD2^–^ and CD2^+^ γδ T cells. CD2^–^ and CD2^+^ γδ T cells were analyzed for their expression of CD8α and CD27. **(A)** Within CD2^–^ γδ T cells CD8α^+/–^CD27^+^ and CD8α^+^CD27^–^ cells were gated. Representative pseudocolor plots for maternal (left) and fetal compartments (right) are shown [applies also for **(D)**]. **(B,C)** Frequencies of CD8α^+/–^CD27^+^ CD2^–^ γδ T cells **(B)** and CD8α^+^CD27^–^ CD2^–^ γδ T cells **(C)** within all investigated anatomic locations. **(D)** CD2^+^ γδ T cells were gated for CD8α^–^CD27^+^, CD8α^+^CD27^+^, CD8α^+^CD27^dim^, and CD8α^+^CD27^–^ cells. **(E–H)** Frequencies of the four CD8α/CD27-defined CD2^+^ γδ T cell subsets within the investigated anatomic locations. **(B,C,E–H)** Each colored symbol represents data from one sow for mBld (*n* = 3) or fetuses coming from that sow for ME (*n* = 8), FP (*n* = 9), and fSpln (*n* = 6). The black bars display the mean within the respective anatomic location.

In this study, we also investigated the two CD2-defined γδ T cell subsets for their CD8α/CD27 expression pattern within mBld, ME, FP, and fSpln (Figure 5). Across all anatomic sites, CD2^–^ γδ T cells mainly had a CD8α^+/–^CD27^+^ phenotype (≥60%) whereas a CD8α^+^CD27^–^ phenotype was only found in mBld and ME (mean: 19 and 36.6%, respectively) (Figures 5A–C). However, it should be noted that for the CD8α^+/–^CD27^+^ phenotype identified within the ME we observed again variations among fetuses influenced by the sow, where for sow No. 3 the mean frequency was 83.2% and for the other two sows this was lower than 60%. This difference might be attributed to a combination of animal-to-animal variation and the age (44). In addition, it should be noted that in the fetal compartments, including FP and fSpln, most CD2^–^ γδ T cells did not express CD8α (Figure 5C). Among the CD2^+^ γδ T cells, four distinct CD8α/CD27-defined phenotypes were characterized (CD8α^–^CD27^+^, CD8α^+^CD27^+^, CD8α^+^CD27^dim^, and CD8α^+^CD27^–^) and highlighted distinct differences between the maternal and fetal sites (Figures 5D–H). The maternal compartments were mainly populated by CD2^+^ γδ T cells with a CD8α^+^CD27^–^ phenotype (mean: 47.8 and 75.5% for cells isolated from mBld and ME) while the fetal compartments were mainly populated by a CD8α^–^CD27^+^ phenotype (mean: 38.8 and 71.6% for cells isolated from FP and fSpln) (Figures 5E,H). CD2^+^ γδ T cells with a CD8α^+^CD27^+^ phenotype were nearly undetectable within the ME but accounted for approximately 20% in the other investigated locations (Figure 5F). In addition, CD2^+^CD8α^+^CD27^–^ γδ T cells were also found in the FP although a degree of variation between individual fetuses could be observed (13.3–45.4%). Lastly, γδ T cells with a CD2^+^CD8α^+^CD27^dim^ phenotype were more prominent in the maternal compartments (Figure 5G).

### Characterization of CD8β T Cells

**FIGURE 6:**
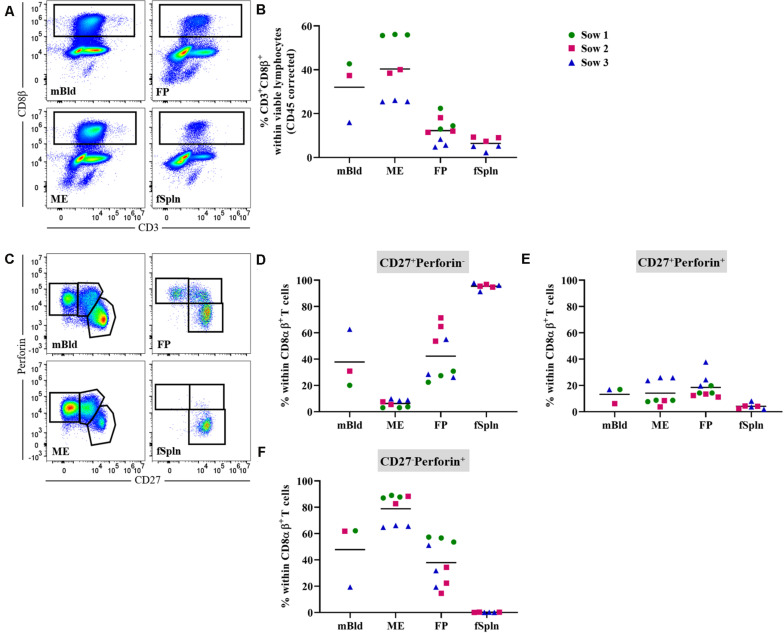
Characterization of porcine CD8 T cells. **(A)** Following exclusion of doublets, dead cells, and cells with high autofluorescence (see Supplementary Figure 1), total CD8 T cells, in the four investigated anatomic locations were identified by gating on CD8β^+^ cells. Representative pseudocolor plots for maternal (left) and fetal compartments (right) are shown [applies also for **(C)**]. **(B)** Frequencies of total CD8β^+^ T cells within viable lymphocytes corrected for CD45 expression. **(C)** Within CD8αβ^+^ T cells (see Supplementary Figure 4) perforin^–^CD27^+^, perforin^+^CD27^+^, and perforin^+^CD27^–^ cells were gated. **(D–F)** Distribution of the three perforin/CD27-defined CD8 T cell populations within the investigated anatomic locations. **(B,D–F)** For all graphs, each colored symbol represents data from one sow for mBld (*n* = 3) or fetuses coming from that sow for ME (*n* = 8), FP (*n* = 9), and fSpln (*n* = 6). The black bars display the mean within the respective anatomic location.

Presently, porcine CD8 T cells can be defined by a CD3^+^CD4^–^CD8α^high^CD8β^+^ phenotype and the expression of perforin in combination with CD27 might be applied to assess differentiation stages (46). Here total CD8 T cells in the anatomic sites from the maternal and fetal compartments were identified by gating on CD3^+^CD8β^+^ cells (Figure 6A). Mean frequencies of CD3^+^CD8β^+^ T cells were higher in the maternal compartments (32 and 40.4% for cells isolated from mBld and ME) as compared to the fetal compartments (12.2 and 6.4% for cells isolated from the FP and fSpln) (Figure 6B). Of note, CD3^+^CD8β^+^ T cell frequencies for sow No. 3 and its fetus-associated tissues (depicted by blue triangle) were consistently lower as opposed to samples from the other sows. Further analysis demonstrated that across all investigated anatomic sites the CD8β^+^ cells co-expressed CD8α and therefore can be regarded as CTLs (Supplementary Figures 4A,B). We assessed the co-expression of perforin and CD27 to gain information about the putative differentiation stages of porcine CTLs. CD27^+^perforin^–^, CD27^+^perforin^+^, and CD27^–^perforin^+^ phenotypes were present within almost all investigated anatomic sites except fSpln (Figure 6C). The frequencies of these CTL subsets for the three sows and fetus-associated tissues are presented in Figures 6D–F. CTLs with a CD27^+^perforin^–^ phenotype were the sole representatives within the fSpln (>95%) and were hardly detected in the ME (mean: 6.2%) (Figure 6D). In mBld and FP the frequency of this phenotype ranged from 20 to 62.6% and 22.4 to 71.3%, respectively. Across all anatomic sites, the abundance of CTLs with a CD27^+^perforin^+^ phenotype was rather low ranging from 4% in the fSpln to 18.5% in FP (Figure 6E). The highest frequency of CTLs with a CD27^–^perforin^+^ phenotype, potentially representing late effectors or memory cells, was found in the ME (mean: 78.9%) followed by mBld (mean: 47.8%) and FP (mean: 37.8%) (Figure 6F).

### Characterization of CD4 T Cells

**FIGURE 7:**
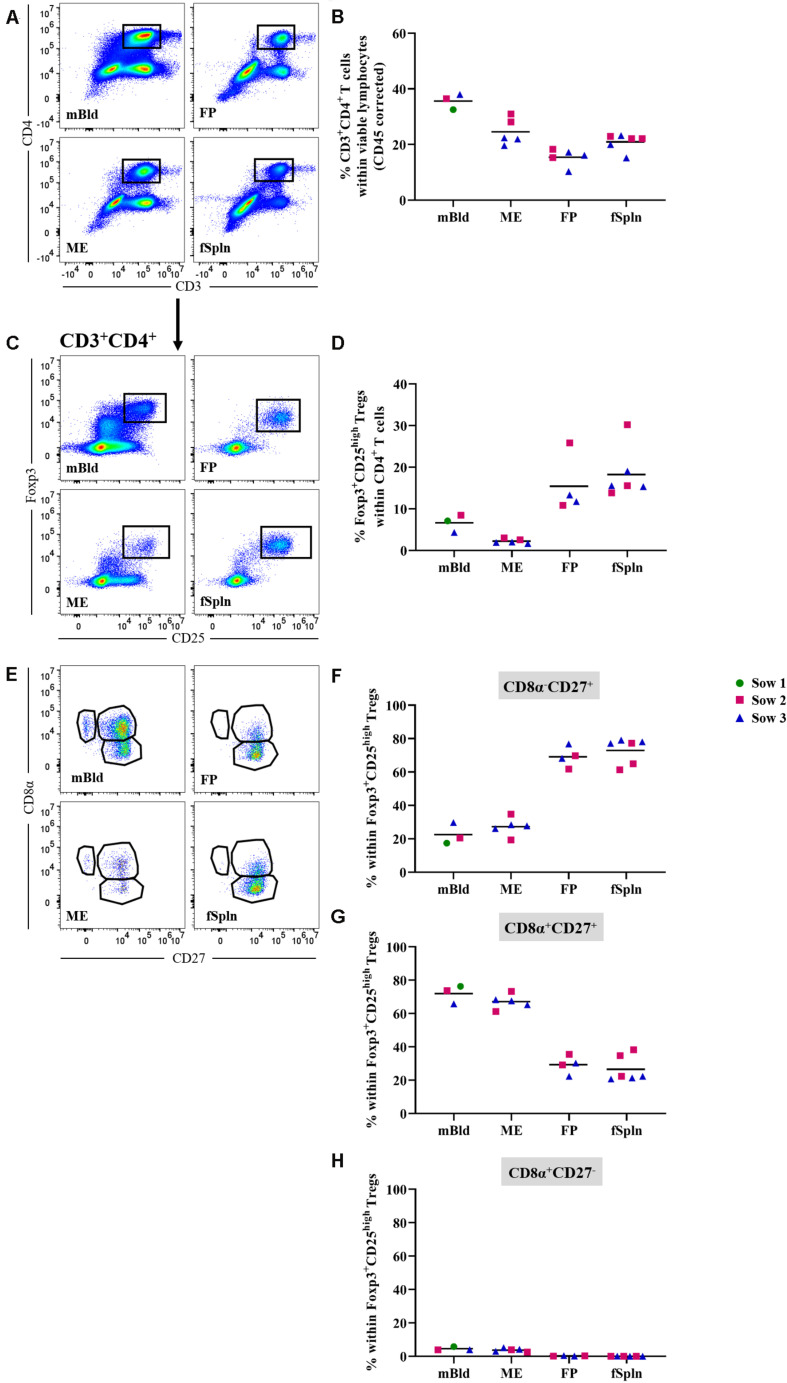
Frequency of total CD4 T cells and characterization of CD4 regulatory T cells. **(A)** Following exclusion of doublets, dead cells, and cells with high autofluorescence (see Supplementary Figure 1), total CD4^+^ T cells were identified by gating on CD3^+^CD4^+^ T cells. Representative pseudocolor plots for maternal (left) and fetal compartments (right) are shown [applies also for **(C,E)**]. **(B)** Frequency of total CD4 T cells within viable lymphocytes corrected for CD45 expression. **(C)** Within total CD4 T cells, Foxp3^+^CD25^high^ cells were gated. **(D)** Frequency of Foxp3^+^CD25^high^ Tregs within total CD4 T cells. **(E)** Foxp3^+^CD25^high^ Tregs were further gated for CD8α^–^CD27^+^, CD8α^–^CD27^+^, and CD8α^–^CD27^+^ cells. **(F–H)** Distribution of the three CD8α/CD27-defined Treg populations. **(B,D,F–H)** For all graphs, each colored symbol represents data from one sow for mBld (*n* = 3) or fetuses coming from that sow for ME (*n* = 5), FP (*n* = 4), and fSpln (*n* = 6). The black bars display the mean within the respective anatomic location.

The differentiation and activation state of porcine CD3^+^CD4^+^ Th cells can be described based on the CD8α/CD27-expression pattern, and transcription factors can be used to address the functionality of these T cells (47–49). In this study we started by investigating frequencies of total Th cells based on a CD3^+^CD4^+^ phenotype (Figure 7A) and the data for total CD4^+^ T cells in the four anatomic sites are displayed in Figure 7. A higher abundance within the maternal compartments (mean: 35.7 and 24.6% for cells isolated from mBld and ME) was found as opposed to less than 20.9% in fetal compartments (mean: 15% in FP and 20.9% in fSpln). Moreover, CD3^+^CD4^+^ T cells were further analyzed for their expression of activation markers, differentiation markers, and transcription factors as outlined below.

#### Regulatory CD4 T Cells

First, we investigated all anatomic sites for the presence and abundance of Tregs based on the expression of Foxp3 and CD25 within CD3^+^CD4^+^ T cells (Figure 7C). Mean Treg frequencies within total CD4^+^ T cells were highest in fSpln and FP with 18.2 and 15.4%, respectively (Figure 7D). The mean Treg frequency in blood-derived CD4^+^ T cells ranged from 4.3 to 8.5% (Figure 7D). More intriguingly, even fewer Tregs could be identified in the ME (2.2%) (Figure 7D). Across all anatomic sites, we further analyzed the CD3^+^CD4^+^Foxp3^+^CD25^high^ Tregs for the expression of CD8α and CD27 which revealed the existence of CD8α^–^CD27^+^, CD8α^+^CD27^+^, and CD8α^+^CD27^–^ Treg phenotypes. However, the latter phenotype was completely absent within the fetal compartments (mean: 0.3% in FP and 0.04% fSpln) and only a few CD8α^+^CD27^–^ Tregs could be observed in mBld and ME (mean: 4.5 and 3.7%, respectively) (Figure 7E). The mean frequencies of the three CD8α/CD27-defined Treg populations are summarized in Figures 7F–H and show that most Tregs in mBld and ME had a CD8α^+^CD27^+^ phenotype (61.3–76.3%) whilst in the FP and fSpln this frequency ranged from 20.6 to 38.2%. Tregs in FP and fSpln mainly displayed a CD8α^–^CD27^+^ phenotype (between 61.3 and 79.1%).

#### Non-regulatory CD4 T Cells

**FIGURE 8:**
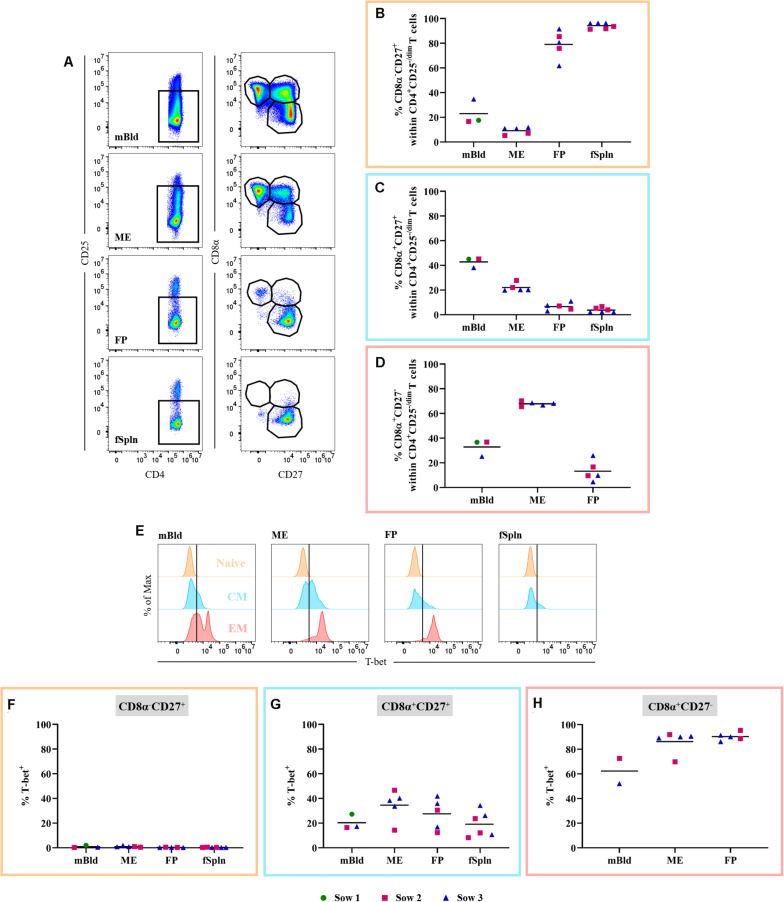
Phenotype and T-bet expression of non-regulatory CD4 T cells. **(A)** Within CD3^+^CD4^+^ T cells CD25^high^ expressing cells were excluded. These cells were gated for CD8α^–^CD27^+^ (representing naive), CD8α^–^CD27^+^ (central memory; Tcm), and CD8α^–^CD27^+^ (effector memory; Tem) cells. Representative pseudocolor plots for maternal and fetal compartments are shown (top to bottom). **(B–D)** Frequency of CD8α/CD27-defined populations within the investigated anatomic locations. **(E)** Non-Treg CD4 T cell populations defined in **A** (naive, Tcm, and Tem) were further analyzed for their expression of the transcription factor T-bet. Representative histograms show the expression of T-bet within the different investigated anatomic locations. **(F,G)** Frequencies of T-bet^+^ cells in naive, Tcm, and Tem within the investigated locations. **(B–D,F–H)** For all graphs, each colored symbol represents data from one sow for mBld (*n* = 3) or fetuses coming from that sow for ME (*n* = 5), FP (*n* = 5), and fSpln (*n* = 6). The black bars display the mean within the respective anatomic locations.

CD3^+^CD4^+^ T cells (Figure 7A) were also investigated in detail for the abundance of non-Treg CD4^+^ T cells within the different anatomic sites. Therefore, we excluded CD4^+^CD25^high^ expressing T cells, the prospective Tregs [Figure 7C and (48)]. Subsequently, CD4^+^CD25^–/dim^ cells were analyzed for the expression of CD8α and CD27 (Figure 8A) delineating a CD8α^–^CD27^+^ naive, CD8α^+^CD27^+^ central memory (Tcm), and CD8α^+^CD27^–^ effector memory (Tem) population. The mean and individual frequencies of these phenotypes for all investigated locations are shown in Figures 8B–D. Again, CD8α^–^CD27^+^ naive CD4^+^ T cells constituted the major fraction in FP and fSpln and only a few antigen-experienced CD4^+^ T cells, with a CD8α^+^CD27^+^ Tcm or CD8α^+^CD27^–^ Tem phenotype, could be identified in the FP. As expected, most non-Treg CD4^+^ T cells isolated from mBld and ME had an antigen-experienced phenotype, hence the high prevalence of CD8α^+^CD27^+^ Tcm in the mBld (mean: 42.8%; Figure 8C) and CD8α^+^CD27^–^ Tem in the ME (mean: 67.8%; Figure 8D). Next, we assessed the Th1 polarization of the three CD8α/CD27-defined CD4^+^ T cells subsets. For each investigated anatomic location, the T-bet expression for CD8α^–^CD27^+^ naive, CD8α^+^CD27^+^ Tcm, and CD8α^+^CD27^–^ Tem populations are depicted in the histograms (Figure 8E). Across all anatomic sites, CD8α^–^CD27^+^ naive CD4^+^ T cells did not express T-bet (Figure 8F), and only a minor portion (∼20%) in mBld and fSpln of the CD8α^+^CD27^+^ Tcm could be identified as T-bet^+^ (Figure 8G). At the maternal-fetal interface, up to 46.6 and 41.9% of the Tcm expressed T-bet (Figure 8G). In mBld, on average 61.7% of the circulating CD8α^+^CD27^–^ Tem cells had a Th1 phenotype whereas in ME and FP most cells expressed T-bet (mean: 86.2 and 90.2%, accordingly) (Figure 8H).

### IFN-γ Production of Lymphocytes at the Maternal-Fetal Interface

**FIGURE 9:**
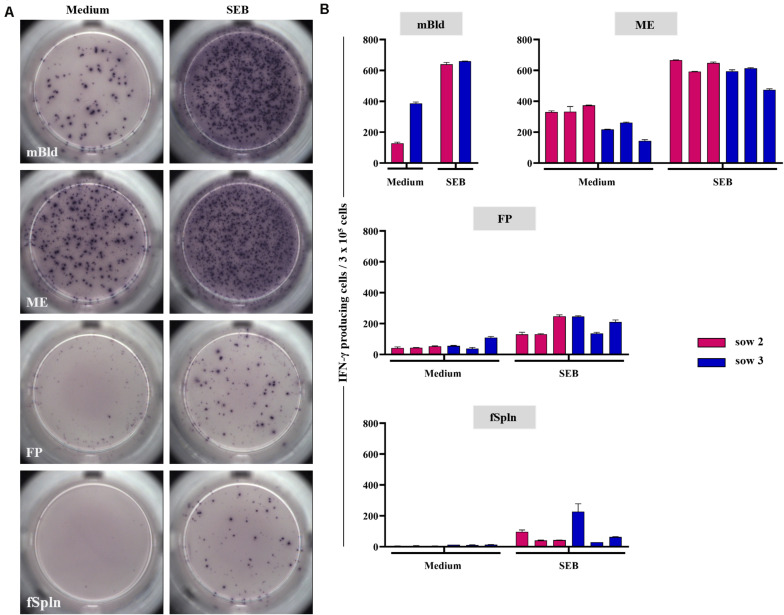
IFN-γ production in ELISpot assays. Lymphocytes isolated from the different anatomic locations were stimulated overnight with staphylococcal enterotoxin B (SEB; 500 ng/mL) or were left untreated. **(A)** Representative raw data for one sow and associated fetal samples. Left: IFN-γ producing cells in unstimulated samples, right: IFN-γ producing cells after SEB stimulation. **(B)** Average IFN-γ producing cells within 3 × 10^5^ cells of two technical replicates for medium control and SEB stimulated cells within each investigated anatomic location. The error bars show the standard error of the mean.

In addition to the phenotyping data, we addressed the capacity for IFN-γ production for the isolated lymphocytes by means of an ELISpot. Following overnight cultivation in the presence of SEB or medium, IFN-γ-producing cells were visualized and quantified. Representative data of IFN-γ-producing cells for one sow and tissues from one associated fetus for medium control or SEB stimulation are presented in Figure 9A (medium, left column; SEB, right column). Results for all sows and all investigated anatomic sites are given in Figure 9B. Maternal lymphocytes, originating from mBld and ME, showed a high but animal-dependent spontaneous IFN-γ secretion following incubation with cell culture medium (Figure 9A, left column and Figure 9B, middle and bottom panel). Spontaneous secretion of IFN-γ for lymphocytes isolated from the FP and fSpln was rather low to non-existent (Figure 9A, left column and Figure 9B, two graphs below) as compared to the maternal lymphocytes. Following stimulation with SEB, IFN-γ-producing cells were observed within all anatomic sites. However, the response was more vigorous for lymphocytes of maternal origin (mBld and ME) in comparison to lymphocytes from FP and fSpln which is in agreement with the increased frequency of memory T cells in the maternal compartments.

## Discussion

So far, research addressing reproductive immunity in the pig is extremely limited. The unique anatomic structure of the porcine placenta prompted us to establish a method that allowed us to elucidate immune cell phenotypes in the maternal and fetal compartment separately. To our knowledge, this has not been attempted before and our methodology will help to investigate immune cell phenotypes present at the maternal-fetal interface. Indeed, in-depth immune phenotyping of NK and T cells highlighted strong differences between ME and FP cell preparations which indicates that the separation contributes to a better understanding of the interplay between the maternal and fetal immune system. Obtaining these unique samples is not that easy, hence, the small sample size, which is one of the limitations in this study. Since this was a first attempt, further research is needed to validate our findings. Furthermore, we were the first to perform these detailed phenotyping experiments and we believe that the groundwork of this study will help to explore lymphocyte function as well as the interplay between pathogens and the immune system *in utero*.

Previous studies showed that porcine NK cells are present in different anatomic locations (e.g., blood, lymphatic, and non-lymphatic organs) (41, 42). Two studies reported an enrichment of CD16^+^ cells in the porcine endometrium during early gestation as opposed to in peripheral blood (37, 38). In our study a more extended NK cell phenotype was used. We showed that total NK cells, with a CD3^–^CD8α^+^CD16^+^CD172a^–^ phenotype, were enriched at the maternal-fetal interface as opposed to the low frequency in the mBld and fSpln. Human and murine NK cells, including uNK cells, express the activating receptor NKp46 (13). In swine, NKp46 delineates three distinct NK cell subsets, namely NKp46^–^, NKp46^+^, and NKp46^high^ (42, 43), which were identified across all investigated anatomic locations in this study. The NKp46^high^ NK cell subset was substantially enriched in the fSpln while being a rarity in the mBld. Our results showed that the maternal-fetal interface is mainly populated by NKp46^–^ and NKp46^+^ NK cells. Recent data suggests that in porcine NK cells the expression of NKp46 gets downregulated during the differentiation process and coincides with a shift in transcription factor expression from Eomes to T-bet (Schmuckenschlager et al., in preparation). On the other hand, NKp46^high^ NK cells have been shown to be the superior source with regard to cytokine production and cytolytic activity (43). Similar to human uNK cells (13), we demonstrated that all identified NK cells contained perforin and, therefore, might possess cytolytic potential. However, the lytic activity of human uNK cells is actively regulated by the expression of human leukocyte antigen (HLA) ligands on the trophoblasts (13), and similar mechanisms might be employed by the porcine placenta. We also showed that prior to birth, fetal NK cells readily express perforin, potentially providing a first line of defense. In conclusion, our data indicates that the porcine maternal-fetal interface is rich in cells with a typical NK cell phenotype but, what this means in terms of local NK cell function remains to be elucidated.

NK cell-associated receptors have been shown to be expressed on a fraction of human decidual T cells at term and might serve as a way to regulate T cell function (19). Furthermore, natural killer T (NKT) cells (50, 51) and mucosal-associated invariant T (MAIT) cells (52) have been shown to populate the human decidua. NKT cells have been linked with pregnancy loss and preterm labor whereas MAIT cells seem to play a role in the anti-bacterial defense (50–52). In our study we identified a population of CD3^+^CD8α^+^CD16^+^CD172a^–^ cells which was enriched in the ME but completely absent in the fSpln. In addition, we also showed that a fraction of these cells expressed NKp46 and all cells were positive for perforin, indicative of cytolytic potential. Within this fraction of CD3^+^CD8α^+^CD16^+^ T cell subsets of CD8β^+^ T cells, NKT cells, MAIT cells, and γδ T cells might be present (41, 53, 54). Nevertheless, our results show that lymphocytes with a mixed NK-/T cell phenotype are localized at the maternal-fetal interface, which suggests that they might play a role in modulating the immune response locally. Additional studies, however, are needed to specifically address which T cell populations are involved.

Pigs are one of the species, including cattle, sheep, and chicken, where γδ T cells represent a substantial proportion of the total T cell population in the blood circulation and secondary lymphoid organs (44, 55). When considering the expression of CD2, a positive and negative γδ T cell subset can be identified and it was suggested that these are two different lineages that are already established in the thymus (44, 56). Based on the role of transcription factors involved in T cell polarization, a recent study addressing the expression of GATA-3 and T-bet in porcine γδ T cells revealed striking differences between the two lineages (44, 49). A mutually exclusive relationship between the expression of GATA-3 and perforin was described and this related to the CD2^–^ and CD2^+^ γδ T cell phenotypes, respectively (44). In agreement to this, we consistently demonstrated that a CD2^–^ phenotype coincided with a GATA-3^+^perforin^–^ co-expression while a CD2^+^ phenotype was associated with a GATA-3^–/low^perforin^+^ phenotype. In swine, the ratio of CD2^–^ to CD2^+^ γδ T cells changes over time in an age-dependent manner and corresponds with a decrease of the GATA-3 expression (44). This is in line with our findings regarding GATA-3 in the CD2^–^ γδ T cells, where the MFI was highest in the fSpln and lowest in the ME. In the mBld and ME, putative effector cells with a CD2^–^perforin^–^CD8α^+^CD27^–^ phenotype were observed and might be a local source of IL-17A (55). Similar observations were found for the CD2^+^ γδ T cells. CD2^+^perforin^+^CD8α^+^CD27^–^ γδ T cells prevailed in the maternal compartments and this phenotype suggests a late stage of differentiation. Surprisingly, this phenotype of putative cytolytic effector cells could also be observed in the FP. Nevertheless, CD2^+^perforin^–^CD8α^dim/–^CD27^+^ γδ T cells dominated the fetal tissues, which appears to correspond to a more naive phenotype. Overall, the γδ T cell composition differed greatly between the maternal and fetal tissues and needs to be investigated further in order to determine the role of γδ T cell subsets at the maternal-fetal interface.

Since CD8α in pigs is abundantly expressed on different immune cell types, a CD3^+^CD8α^high^CD8β^+^ phenotype was investigated to identify the abundance of CTLs within all investigated locations. The phenotypic differentiation of these cells in the pig is not yet completely elucidated; however, studies suggest that the combination of perforin and CD27 expression can be applied to assess CTL differentiation (46, 57, 58). Our results showed that in the fSpln all CTLs have a perforin^–^CD27^+^ phenotype. This validates the findings that porcine neonates were born with naive CTLs (46). Over time these cells acquired the expression of perforin which coincides with a down regulation of CD27 (46). Perforin^+^CD27^+^ might represent an early effector or Tcm phenotype while the complete loss of CD27 (perforin^+^CD27^–^) might indicate a terminally differentiated phenotype and, therefore, might contain late effectors or Tem cells (46, 57). Similar to human dCD8^+^ T cells (22, 23, 26, 30), we found that CTLs with a putative Tcm and Tem phenotype were enriched in the maternal compartments. To our surprise, we also detected a substantial proportion of CTLs with a Tcm and Tem phenotype in the FP. Currently, we do not know if the enrichment of antigen-experienced cells in the FP might be explained by a migration of maternal cells to the FP or if they represent antigen-experienced T cells of fetal origin.

Considering that CD4^+^ T cells can be identified in the human decidua (8, 27), we also characterized CD3^+^CD4^+^ T cells at the porcine maternal-fetal interface, the mBld, and fSpln. Surprisingly, our in-depth analysis revealed that the Treg frequency in the ME was extremely low whereas an enrichment was observed in both fetal tissues. Although the abundance of Tregs was low in the maternal compartments, the majority was CD8α^+^. Upregulation of CD8α on porcine CD4^+^ T cells coincides with antigen-experience and is associated with immunological memory [reviewed in (57)], hence, this might indicate that ME Tregs are activated or in a memory-like state. Differently, in the fetal compartments most Tregs did not express CD8α, indicating a more naive state. Furthermore, analysis of non-Tregs showed that differentiated phenotypes, including Tcm and Tem, prevailed in mBld and ME, which is in agreement with results from the human field (27). As expected, the non-Treg CD4^+^ T cells from the fetal tissues were predominantly naive, aside from the small Tem population in the FP. In pigs, the characterization of polarized CD4^+^ T cell subsets is not as straightforward because not all subsets have been identified so far (57). However, it has been shown that following an infection with PRRSV an increase in T-bet^+^ CD4^+^ T cells can be observed (59). Evidently, a recent study demonstrated that also T-bet expression of porcine αβ T cells is associated with IFN-γ production (49). In the current study, the vast majority of Tem cells at the maternal-fetal interface, regardless of the anatomic site, expressed T-bet. In context of human pregnancy, a recent study demonstrated that PBMCs gradually acquire a more activated phenotype following the transition from the second to third trimester, but are still regulated (3). This transition is necessary for parturition (2). In our study the low abundance of Tregs in the ME might be an indicator of a gradual lift of the local immunosuppressive regulation in order to prepare for parturition. In line with this, it has been shown that the suppressive activity of Tregs decreases at term and thereby is implicated in the induction of parturition (20). However, investigating Treg frequencies and function at different gestational stages is necessary to address these speculations. Initially, pregnancy has been defined as being a Th2 phenomenon; however, first and last trimester decidua are enriched with Th1 cells (8, 27). In line with this, our data indicates that most terminally differentiated CD4^+^ T cells have a Th1 phenotype at the porcine maternal-fetal interface. Prior to parturition, these cells might create a type-1 cytokine environment. Moreover, human CD4^+^ T cells at the maternal-fetal interface have been shown to produce a variety of pro-inflammatory cytokines and matrix metalloproteinase-9 by which they play a role in the onset and perpetuation of parturition (20). Hence, the T-bet^+^ Tem cells identified at the maternal-fetal interface in our study might have a similar function.

Overall, we identified several T cell populations, e.g., CD2^+^perforin^+^CD8α^+^CD27^–^ γδ T cells, perforin^+^CD27^–^ CTLs, and T-bet^+^ CD4^+^ Tem cells, with putative effector functions in the FP. In humans and mice, microchimerism is a well-established fact. Therefore, these putative effector cells might be maternal cells that have migrated to the FP. However, the porcine placenta is considered as a tight impermeable barrier and contrasting findings regarding microchimerism in pregnant pigs have been reported (60, 61). Female DNA was found in the serum of male fetuses and female cells were detected in the male fetal liver, but the origin of the DNA and cells, either maternal or female siblings, could not be tracked (60). Furthermore, male DNA was detected in the maternal circulation (60). Data from another group, contradicts the previous findings and showed that there was no exchange of cells (61). It is also plausible that the effector cells in the FP are of fetal origin; however, the cues that drive their differentiation remain to be elucidated. Different pathogens, e.g., porcine parvovirus, manage to breach the placenta or reside in the uterus. Thus, the presence of local pathogens during late gestation might be implicated in the differentiation of effector cells.

The functional capacity of our isolated cells was assessed by means of an IFN-γ ELISpot assay. In the human field, SEB stimulation is often used as a positive control to induce cytokine production in T cells (62–64) and therefore was applied in our study. Our results demonstrated a substantial spontaneous IFN-γ release by cells isolated from the mBld and ME, which reflects the presence of highly differentiated cells within these anatomic sites. Lymphocytes isolated from all anatomic sites were able to produce IFN-γ, but the response for the fetal compartments was limited, further demonstrating that the fetal compartments reflect a naive immune phenotype in general.

In conclusion, with our uniquely designed methodology and the available porcine toolbox we were able to reveal immune phenotypes that reside at the maternal-fetal interface. Overall, a naive immune phenotype predominated the fetal compartments as opposed to the antigen-experienced immune phenotype of the maternal system. The physiological role of these cells during gestation and how they are coordinated open a broad array of questions that need to be answered. The groundwork of this study will help to explore lymphocyte function as well as the interplay between pathogens and the immune system *in utero* at different stages of gestation. Such findings might also instruct vaccine development and optimization.

## Data Availability Statement

The raw data supporting the conclusions of this article will be made available by the authors, without undue reservation.

## Ethics Statement

Ethical review and approval was not required for the animal study because no live animals were included. Samples were collected from dead animals which does not require governmental animal ethics approval in Austria. The project plan has been discussed and approved by the institutional ethics and animal welfare committee in accordance with GSP guidelines and national legislation (approval number ETK-32/02/2016).

## Author Contributions

MRS, KM, AS, WG, and AL were in charge of the study design. ES and SS were involved in tissue collection and the tissue separation procedure. MRS, MK, MS, SS, and ES performed laboratory work and experiments. MRS carried out the phenotyping experiments and analyzed the data. MRS, KM, ES, AS, WG, and AL thoroughly discussed and interpreted the data. MRS, WG, and AL wrote the manuscript. All authors read and approved the final manuscript.

## Conflict of Interest

The authors declare that the research was conducted in the absence of any commercial or financial relationships that could be construed as a potential conflict of interest.
