# Supplementary material for: NK and T Cell Differentiation at the Maternal-Fetal Interface in Sows During Late Gestation
Source: Front Immunol. 2020 Sep 11;11:582065. doi: 10.3389/fimmu.2020.582065 (PMC7516083; doi:10.3389/fimmu.2020.582065)
Supplement: Supplementary file 1 [file Table_1.DOCX]

Supplementary Material

NK and T cell differentiation at the maternal-fetal interface in sows during late gestation

Melissa R. Stas, Michaela Koch, Maria Stadler, Spencer Sawyer, Elena L. Sassu, Kerstin H. Mair, Armin Saalmüller, Wilhelm Gerner^†^ and Andrea Ladinig^†*^

^†^These authors have contributed equally to this work.

* Correspondence: Andrea Ladinig: [Andrea.ladinig@vetmeduni.ac.at](mailto:Andrea.ladinig@vetmeduni.ac.at)

# Supplementary Figures

**
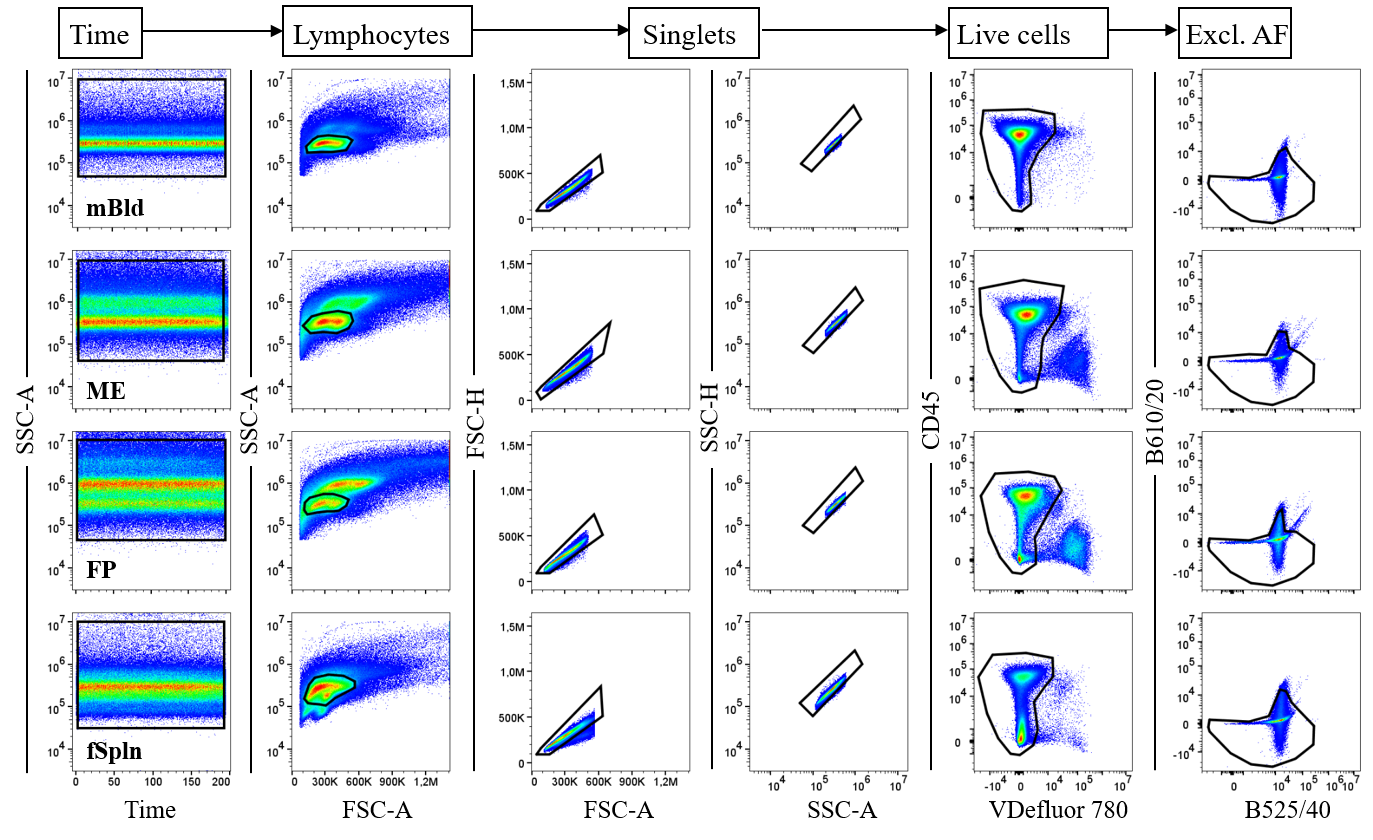
**

**Supplementary Figure 1** **| Consecutive gating strategy for lymphocytes isolated from the investigated anatomic locations.** For each flow cytometry staining panel (see Table 1) the following gating hierarchy was applied: after a time gate, lymphocytes were selected according to their light scatter properties (FSC-A vs. SSC-A). Thereafter, a two-step doublet discrimination (FSC-H vs. FSC-A and SSC-H vs. SSC-A) was applied, which was followed by the exclusion of dead cells, based on a staining with the fixable viability dye eFluor780^®^. Hereafter, cells with a high auto fluorescent signal were excluded by using the bandpass filter 610/20 in the excitation line of the blue laser. Representative pseudocolor plots are shown (top to bottom): mBld, maternal blood; ME, maternal endometrium; FP, fetal placenta; fSpln, fetal spleen.

**
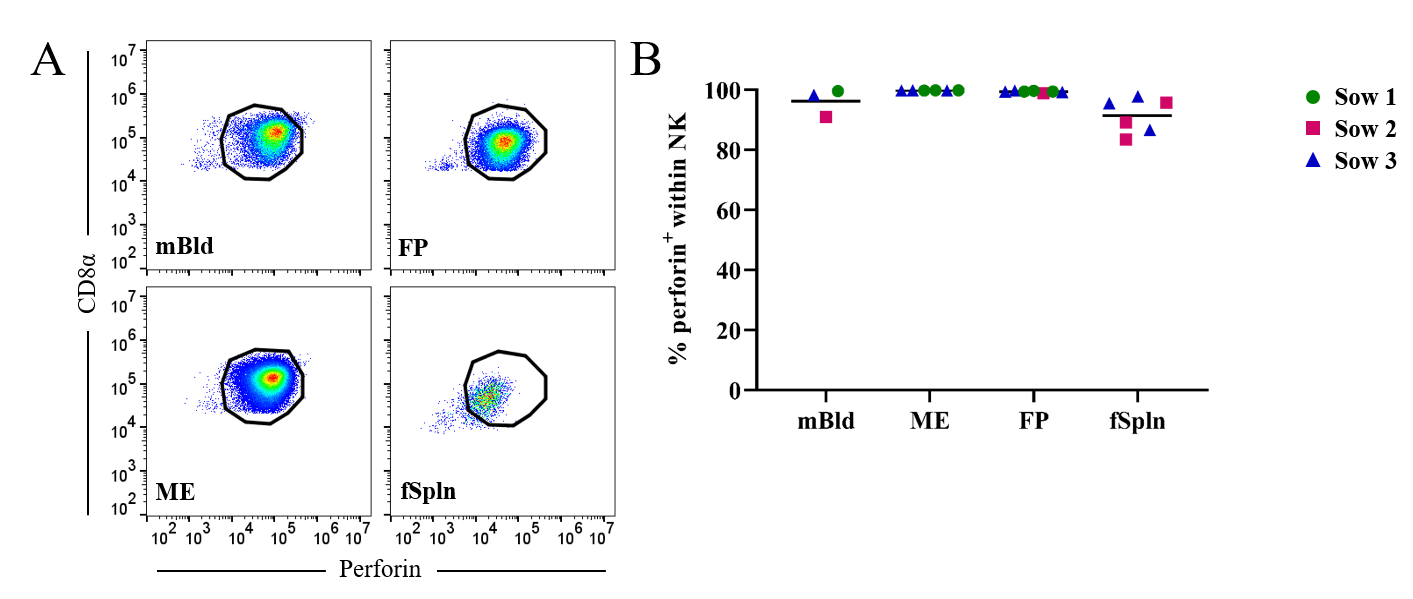
**

**Supplementary Figure 2** **| Expression of perforin in cells with an NK cell phenotype. (A)** CD3^-^CD8α^+^CD16^+^CD172a^-^ cells (see Figure 2A) were further analyzed for their expression of perforin within all investigated anatomic locations. Representative pseudocolor plots for maternal (left) and fetal compartments (right) are shown. **(B)** Frequency of perforin^+^ NK cells (CD3^-^CD8α^+^CD16^+^CD172a^-^) within the respective anatomic locations. Each colored symbol represents data from one sow for mBld (n = 3) or fetuses coming from that sow ME (n = 6), FP (n = 7), and fSpln (n = 6). The black bars display the mean within the respective anatomic location.

**
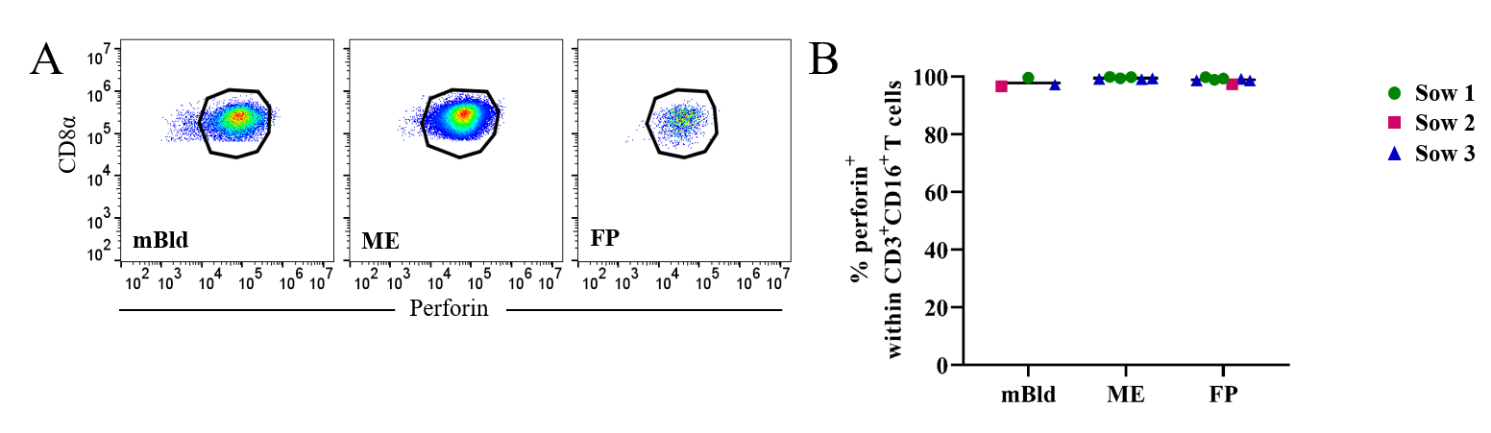
**

**Supplementary Figure 3** **| Expression of perforin in CD3^+^CD16^+^ T cells. (A)** CD16^+^ T cells (CD3^+^CD8α^+^CD16^+^CD172a^-^, see Figure 3A) were further analyzed for their expression of perforin. No CD16^+^ T cells were found in fetal spleens (see Figure 3A) hence this location was not investigated further. Representative pseudocolor plots for maternal (left) and fetal compartments (right) are shown. **(B)** Frequency of perforin^+^ cells within CD3^+^CD16^+^ T cells in mBld, ME and FP. Each colored symbol represents data from one sow for mBld (n = 3) or fetuses coming from that sow ME (n = 6), FP (n = 7), and fSpln (n = 6). The black bars display the mean within the respective anatomic location.

**
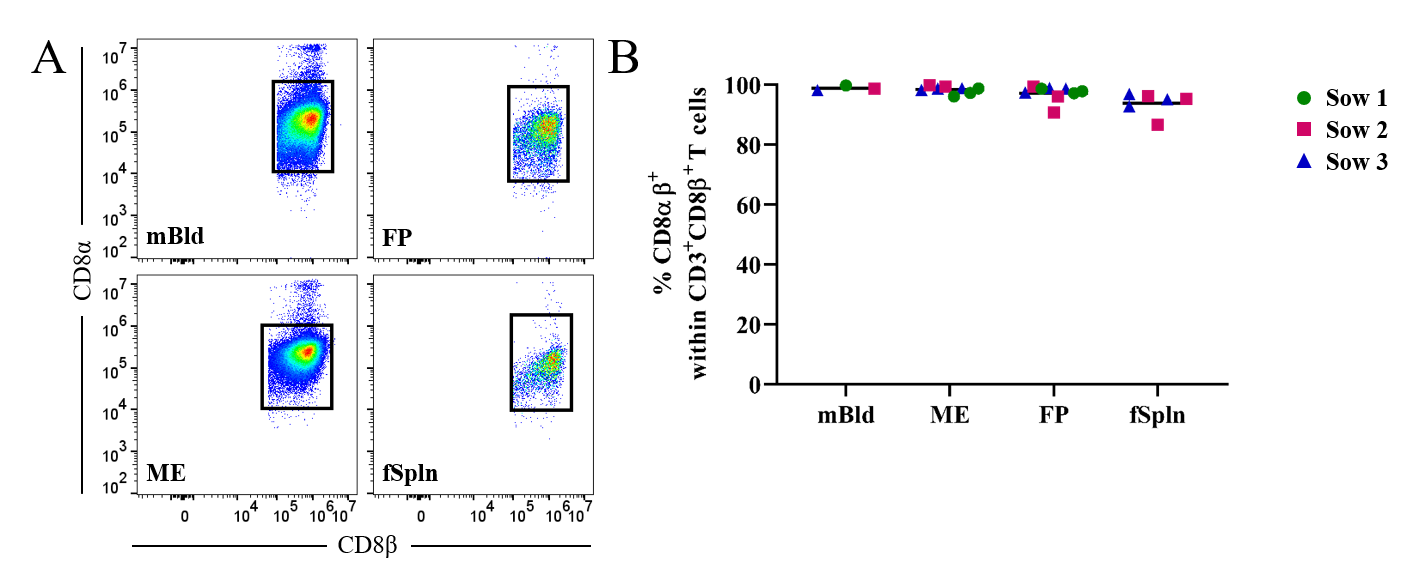
**

**Supplementary Figure 4** **| Expression of CD8αβ heterodimers on CD8 T cells. (A)**, CD8β^+^ T cells (see Figure 6A) were further gated for their co-expression of CD8α and CD8β. Representative pseudocolor plots for maternal (left) and fetal compartments (right) are shown. **(B)** Frequency of CD8αβ heterodimer expressing cells within total CD8β^+^ cells. Each colored symbol represents data from one sow for mBld (n = 3) or fetuses coming from that sow ME (n = 8), FP (n = 9), and fSpln (n = 6). The black bars display the mean within the respective anatomic location.
